# Optimized biosynthesis of bioactive silver nanoparticle-kombucha cellulose nanocomposites for enhanced antimicrobial applications

**DOI:** 10.1007/s00253-025-13585-0

**Published:** 2025-09-29

**Authors:** M. Bassam Aboul-Nasr, Alaa A. Yasien, Sabah S. Mohamed, Marwa Obiedallah

**Affiliations:** Botany and Microbiology Department, Faculty of Science, Sohag University, Sohag, 82524 Egypt

**Keywords:** Fungal biosynthesis, Bacterial cellulose nanocomposites, Antimicrobial nanomaterials, Green synthesis optimization, DOE/RSM modeling, Endophyte-mediated nanoparticle synthesis

## Abstract

**Abstract:**

Sustainable biosynthesis of monodisperse, bioactive silver nanoparticles (AgNPs) integrated with nanofabricated kombucha SCOBY cellulose membrane (KC) was achieved using the endophytic fungus *A*. *fumigatiaffinis* PP235788.1 fungal filtrate. This study is the first to optimize AgNP synthesis via full-factorial design of experiments (DOEs) and response surface methodology (RSM), identifying ideal conditions (2 mmol L^−1^ AgNO₃, pH 8 and 60 °C). Characterization revealed spherical AgNPs (14.50 ± 0.58 nm) and AgNPs@KC composites (17.88 ± 0.36 nm); SEM imaging demonstrates the successful fusion of AgNPs within KC’s fibrous membrane, creating a seamlessly integrated nanocomposite with enhanced functional architecture. The formed nanoparticles were composed of crystalline mettalic silver, as varified by XRD (detected planes at 111, 200, and 220), and were stabilized by amide groups, as identified by FTIR spectroscopy. The biosynthesized AgNPs demonstrated a dose-dependent antimicrobial activity with an increase in inhibition zones from 11.8 ± 2.1 mm at 10 µg/mL to 36.1 ± 0.4 mm at 100 µg/mL for *E*. *coli* and from 9.8 ± 2.9 mm to 34.33 ± 1.2 mm for *Bacillus subtilis*. Fungal pathogens displayed diminished sensitivity, with *Aspergillus niger* achieving maximum inhibition (25.7 ± 0.3 mm) at 100 µg/mL, whereas *Candida* spp. necessitated ≥ 60 µg/mL for observable inhibition zones. AgNPs@KC composites demonstrated broad-spectrum efficacy against 12 pathogens (ANOVA, *p* < 0.001, *R*^2^ > 95%), with maximal activity against *E*. *coli*, *B*. *subtilis*, and *A*. *niger* (33.0 ± 0.2 mm). These findings highlight the integration of mycosynthesized AgNPs with kombucha SCOBY cellulose membranes, offering a novel and unique platform for antimicrobial approaches with exceptional purity and distinctive physiological characteristics.

**Key points:**

• *AgNPs optimized via DOE/RSM.*

• *AgNPs@KC shows potent antimicrobial activity.*

• *Novel mycosynthesis of AgNPs with KC membranes.*

**Supplementary Information:**

The online version contains supplementary material available at 10.1007/s00253-025-13585-0.

## Introduction

Recently, natural biopolymers such as gelatin, chitosan, and albumin have attracted increased interest in the field of research because of their potential in the field of biomedicine (da Silva et al. 2010; Rajwade et al. 2015). Among these biopolymers, cellulose has garnered significant attention because of its abundant availability and distinctive physiological characteristics. A primary limitation of plant-derived cellulose in medical and pharmaceutical contexts is its impurity, necessitating thorough pretreatment procedures prior to utilization (Shah et al. 2013). Therefore, specific microorganisms have been recognized for their ability to produce cellulose from various natural and artificial sugar substrates. This form of cellulose, known as microbial cellulose or bacterial cellulose (BC), offers exceptional purity and addresses the challenges associated with plant-derived cellulose (Ul-Islam et al. 2019). The kombucha SCOBY (symbiotic culture of bacteria and yeast) produces a specialized type of BC, often referred to as kombucha cellulose (KC), which shares the fundamental properties of BC but is synthesized by a unique microbial consortium (Acetobacter spp. and yeasts) during tea fermentation (Jayabalan et al. 2014; Villarreal-Soto et al. 2018). Bacterial cellulose is a compelling material for wound dressings due to its ability to create a moist wound environment that promotes healing. This occurs through BC’s highly porous 3D nanofibrillar network (with pore diameters of 20–100 nm), which maintains optimal moisture retention while allowing gas exchange—critical for tissue regeneration (Klemm et al. 2005; Barud et al. 2007). Furthermore, the high water-holding capacity (up to 200 times its dry weight) prevents wound desiccation while absorbing exudate, and the nanostructured surface mimics the extracellular matrix to support cell migration (Ul-Islam et al. 2019).

Kombucha, a fermented, mildly alcoholic beverage, is commonly produced by utilizing sweetened black or green tea. It is sometimes referred to as kombucha tea to distinguish it from the specific bacteria and yeast culture involved in the fermentation process. The symbiotic culture of bacteria and yeast (SCOBY) present in kombucha can exhibit variations in microbial populations, typically consisting of bacterial species such as *Acetobacter*, various *Saccharomyces* strains, and several other types of yeasts (Jayabalan et al. 2014). Additionally, *Acetobacter* can polymerize glucose, forming bacterial cellulose that supports microbial communities (Jayabalan et al. 2014; Villarreal‐Soto et al. (2018). Owing to its high crystallinity, stiffness, and numerous advantages, BC has recently garnered considerable interest for its potential in various industrial uses (Soemphol et al. 2020; Menon et al. 2017). One promising area of research involves combining BC with silver nanoparticles (AgNPs) (Yang et al. 2012; Maneerung et al. 2008). Silver nanoparticles exhibit unique optical properties, including vivid color changes (e.g., yellow to brown) upon formation, due to their interaction with light—a phenomenon known as surface plasmon resonance (Kelly et al. 2003). These visual characteristics serve as key indicators of nanoparticle synthesis and stability. Additionally, AgNPs exhibit potent antimicrobial activity through multiple mechanisms, including membrane disruption, reactive oxygen species (ROS) generation, and interference with DNA replication—collectively leading to microbial cell death (Fayaz et al. 2010).

While various methods exist for synthesizing metal nanoparticles, chemical approaches often generate toxic byproducts (e.g., borohydride residues, citrate derivatives), and physical methods require high energy consumption—both posing substantial environmental risks (Maneerung et al. 2008; Sharma et al. 2009). Consequently, green nanotechnology—utilizing biological pathways like microorganisms and plants for nanoparticle synthesis—has emerged as a viable alternative, offering greater environmental sustainability and cost-effectiveness compared to conventional methods (Patra & Baek 2014). Fungal systems offer distinct advantages over plant and bacterial synthesis methods, including higher secretion of reducing enzymes (e.g., NADH-dependent reductases), superior metal ion tolerance, and easier biomass handling for large-scale production. Their dense mycelial networks provide extensive surface area for metal ion adsorption, while extracellular proteins enable efficient stabilization of nanoparticles without additional capping agents (Sidhu et al. 2025). Compared to plant extracts (which show seasonal variability) and bacteria (with lower yield), fungi consistently produce monodisperse nanoparticles with exceptional stability, as demonstrated in recent mycogenic synthesis studies (Sudheer et al. 2022).

Endophytic fungi represent an eco-efficient alternative for nanoparticle biosynthesis, leveraging their intrinsic plant-associated symbiosis and metal-reducing extracellular enzymes. These microorganisms naturally produce NPs with enhanced bioactivity profiles (antimicrobial/antioxidant) while demonstrating scalable cultivation and inherent stress tolerance—attributes that position them as ideal candidates for developing nano-enabled agricultural solutions targeting crop protection and yield enhancement under climate change scenarios (Adeleke et al. 2024).

Fungal systems are pivotal for green nanoparticle synthesis, offering superior scalability and enzyme diversity compared to bacteria or plants (Verma et al. 2019). Among fungi, the endophyte *A*. *fumigatiaffinis* was selected for its exceptional metal tolerance and reductase activity, as demonstrated in our prior work (Obiedallah et al. 2024). Comparative studies with common fungal strains (e.g., *A*. *niger*, *F*. *oxysporum*) confirm its superior AgNP synthesis rates and monodispersity under identical conditions (Birla et al. 2013; Verma et al. 2019). Previous research has demonstrated the impregnation of AgNPs into BC through chemical reduction with a silver nitrate solution (Jayabalan et al. 2014). Various reducing agents, including borohydride, citrate, ascorbate, hydrazine, and hydroxylamine, are commonly employed to convert absorbed silver ions within BC to metallic AgNPs (Maneerung et al. 2008; de Santa Maria et al. 2009; Sharma et al. 2009). However, these agents may pose environmental or biological risks. Consequently, there is a growing need for eco-friendly and sustainable methods to produce AgNPs in BC for medical applications, such as the use of benign solvents and non-toxic materials (Patra and Baek 2014).

While previous studies have explored the synthesis of AgNPs and their integration with bacterial cellulose, the combination of mycosynthesized AgNPs with the kombucha SCOBY cellulose membrane remains unexplored. This study presents a novel approach by involving the use of the endophytic fungus *A*. *fumigatiaffinis* PP235788.1 for AgNP biosynthesis and its immobilization on kombucha SCOBY cellulose, offering a unique platform for antimicrobial applications.

## Materials and methods

### Source of strains

In our previous study, *Aspergillus fumigatiaffinis* PP235788.1, an endophytic fungus isolated from the roots of *Artemisia judaica* (Obiedallah et al. 2024), was selected as a novel source for synthesizing AgNPs. All the microbial strains utilized in the assessment of antimicrobial efficacy were obtained from the Mycology and Bacteriology Laboratories, Department of Botany and Microbiology, Faculty of Science at Sohag University.

### Preparation of broth and culture of *Aspergillus fumigatiaffinis* PP235788.1

The broth was prepared for the culture of *A*. *fumigatiaffinis* PP235788.1 following standardized mycological protocols for endophytic fungi (Sidhu et al. 2025), with modifications: potato dextrose broth (24 g/L) adjusted to pH 6.5 before autoclaving. Three 250-mL conical flasks, each containing 100 mL of potato dextrose broth (PDB) (boiling 0.2 kg of potatoes per Liter for 30 min and passing through cheesecloth), were used to create a potato extract infusion. Subsequently, 0.02 kg per liter of dextrose (pH 6.5) was added, and the mixture was autoclaved at 20 psi for 20 min. *Aspergillus fumigatiaffinis* colonies grown on Czapek’s Dox agar plates were inoculated by transferring five 6-mm mycelial plugs (from actively growing colony margins) into each flask. For 8 days, the conical flasks were incubated at 28 ± 1.0 °C.

### Biomass preparation

Following incubation, the mycelial mat was filtered out of the media, and any remaining medium components were thoroughly removed from the biomass via sterile deionized water. To induce extracellular enzyme secretion (e.g., NADH-dependent reductases critical for Ag⁺ reduction), fungal biomass (10 g, fresh weight) was incubated in deionized water (100 mL) for 72 h in darkness (28 ± 1.0 °C, on a rotary shaker, 120 rpm) to prevent photodegradation of redox-active metabolites, as demonstrated in prior fungal nanosynthesis studies (Birla et al. 2013; Aboul-Nasr et al. 2021). Sterile Whatman filter paper no. 1 was used to separate the fungal mat from the fungal-secreted enzymes in the filtrate (Ahmad et al. 2003).

### Biosynthesis of silver nanoparticles

Silver nanoparticle synthesis was performed by reacting 2 mL of 10 mM AgNO₃ (ITW Reagents, #131,459; ACS grade, purity ≥ 99.8%) with 18 mL of fungal filtrate (final 1 mmol L^−1^ AgNO₃) at pH 8.0 (adjusted with 0.1 M NaOH) and 60 °C for 24 h in the dark. pH and temperature were monitored using a calibrated pH meter (Hanna Instruments, HI2210) and digital thermometer (VWR, #61,144–582). The initial AgNO₃ concentration (1 mmol L^−1^ final) was selected based on its optimal balance between nanoparticle yield and fungal viability, as lower concentrations (< 0.5 mmol L^−1^) produced insufficient nanoparticles, while higher concentrations (> 3 mmol L^−1^) induced cellular stress (de Silva 2024). The subsequent step involved the incubation of the test tubes at 25 ± 1.0 °C for a Duration of 24 h under conditions devoid of light to prevent any potential photochemical reactions that could impact the outcome of the experiment. The fungal filtrate was subjected to identical environmental conditions to serve as an experimental control. The process of silver reduction was monitored through visual examination, whereby the supernatant transitioned from a light yellow hue to a brown color, serving as a practical indicator of the synthesis of AgNPs. The UV–visible spectra of the samples were analyzed to investigate AgNP formation. After the reaction, the colloidal solution was centrifuged at 10,000 rpm (≈9300 × g) for 15 min at 4 °C using a fixed-angle rotor (Centurion Scientific K241R, swing-out rotor BRK5510), consistent with standard nanoparticle recovery protocols (Sudheer et al. 2022). The pellet was washed three times with sterile distilled water (resuspended by pipetting and gentle vortexing), then air-dried at 25 °C in the dark.

### Optimizing AgNPs production via *Aspergillus fumigatiaffinis* PP235788

Using SigmaXL Version 10, a design of experiment (DOE) statistical analysis was conducted to assess the impact of three variables on the production of AgNPs. A two-level factorial design experimental matrix (16-run, full factorial) was established on the basis of three variables: AgNO₃ concentration, pH, and temperature. Two levels, “low” and “high,” were assigned to each factor to assess the response (optical density (OD) at 425 nm) of AgNP production after 24 h). The response (OD) correlates with and is proportional to the concentration and size of the formed nanoparticles. The settings for the Ag^+^ concentration, pH, and temperature were “1, 2, and 3 mmol L^−1^”; “6, 7, and 8”; and “20, 40, and 60,” respectively (Table 1). All tests were carried out in 20 mL (final volume), with two biological replicates used to increase the standard deviation value and a randomized experimental run sequence to reduce the impact of uncontrollable variables. Unless explicitly indicated in the design of experimental (DOE) matrix, the experimental parameters were set to pH 6.5, 1 mM AgNO₃, and 28 ± 1.0°C. Post-production, the optical density of the AgNPs was quantified via UV–visible spectroscopy. The optimal conditions for AgNP production revealed that the AgNP concentration and pH were the most influential factors. As a result, these two parameters were chosen for response surface methodology (RSM) analysis. RSM is an effective approach for optimizing microbial production and developing statistical models that use several parameter combinations to forecast the response variable. A central composite design (CCD) with two central points was employed, including two replicates, and the axial alpha value was set to face-centered (*α* = 1.0) for a total of 20 experimental runs (Table 2). Three center points—low, medium, and high—represent each of the factors in the design used. To determine the impacts of the pH and Ag^+^ concentration on AgNP formation, two-way ANOVA was used to statistically analyze the two generated models, the full-factorial design and RSM Supplementary Table 2 (Online Resource 1).

**Table 1:** Experimental design matrix and results for the 2^3^ full-factorial design for evaluating the effects of factors A, B, and C on AgNP production (OD). The design includes one block and one center point

Run	Std	A	B	C	OD (response)	Predicted OD	Residuals
1	11	1	60	6	1.19	1.10	− 0.01
2	16	3	60	8	1.70	1.73	− 0.01
3	4	3	60	6	1.48	1.50	− 0.01
4	12	3	60	6	1.50	1.50	0.01
5	1	1	20	6	0.78	0.77	0.02
6	8	3	60	8	1.75	1.73	0.03
7	15	1	60	8	1.40	1.34	0.01
8	13	1	20	8	1.20	1.195	0.01
9	14	3	20	8	1.63	1.59	0.01
10	5	1	20	8	1.19	1.16	− 0.01
11	10	3	20	6	1.28	1.30	− 0.01
12	2	3	20	6	1.32	1.31	0.01
13	7	1	60	8	1.39	1.31	− 0.01
14	9	1	20	6	0.75	0.77	− 0.02
15	3	1	60	6	1.22	1.20	0.01
16	6	3	20	8	1.58	1.30	− 0.01

The center point is included to assess the curvature in the model. A: AgNO₃ concentration (mmol L^−1^), B: temperature (°C), C: pH, and OD: optical density at 425 nm

**Table 2:** Optimization of AgNP production (OD) via response surface methodology (RSM): predicted and residual values for AgNO₃ concentration and pH (20 experimental runs)

Run	Std	Center points	A	B	OD (response)	Predicted OD	Residuals
1	12	1	3	6	1.48	1.46	0.02
2	11	1	1	6	1.3	1.29	0.01
3	14	1	3	8	1.78	1.77	0.01
4	15	1	1	7	1.42	1.43	− 0.00
5	16	1	3	7	1.62	1.64	− 0.02
6	13	1	1	8	1.52	1.52	− 0.00
7	17	1	2	6	1.32	1.34	− 0.02
8	18	1	2	7	1.52	1.50	0.02
9	19	0	2	8	1.61	1.62	− 0.01
10	20	0	2	8	1.62	1.62	0.01
11	5	1	1	7	1.42	1.43	− 0.01
12	2	1	3	6	1.48	1.47	0.01
13	9	0	2	8	1.52	1.50	0.02
14	3	1	1	8	1.52	1.52	− 0.00
15	10	0	2	7	1.52	1.50	0.02
16	6	1	3	7	1.62	1.64	− 0.02
17	7	1	2	6	1.32	1.35	− 0.02
18	1	1	1	6	1.29	1.29	0.00
19	8	1	2	8	1.62	1.68	0.00
20	4	1	3	8	1.78	1.78	0.00

A: concentration (°C), B: pH, and OD: optical density at 425 nm

### Growth conditions of the SCOBY disc

The symbiotic culture of bacteria and yeast (SCOBY) disc was produced by fermenting sweetened green tea (100 g sucrose in 1 L boiling deionized water with three commercial tea bags [Amazon US, green tea]) inoculated with a SCOBY (symbiotic culture of *Komagataeibacter* spp. and *Saccharomyces* spp., per Jayabalan et al. 2014) in a sterilized 1-gallon glass jar at 25 ± 1 °C for 7 days. The bacterial cellulose membrane (BCM) was purified by boiling in 1.5% (w/v) NaOH for 2 h to remove microbial cells, followed by rinsing to neutral pH (Yang et al. 2012).

### Purification of the kombucha cellulose membrane

The kombucha cellulose membrane was autoclaved for 20 min. at 20 psi. After that, it was carefully dried with paper after being cleaned six times with 500 mL of deionized water each time. The membrane was then submerged in 50 mL of 1.5% (w/w) aqueous hydrogen peroxide (H₂O₂) and allowed to dry for 2 h at room temperature (25 °C). The 1.5% H₂O₂ treatment served dual purposes: (1) sterilization of the cellulose matrix by oxidative destruction of residual SCOBY microorganisms and (2) functionalization of hydroxyl groups for enhanced AgNPs binding (Yang et al. 2012). Following extraction from the H₂O₂ solution, the membrane was patted dry with Kleenex tissues and thoroughly rinsed six times with 500 mL of sterile deionized water each time.

### Production of the AgNPs@KC nanocomposites

The kombucha cellulose membrane was functionalized with a solution that included powdered AgNPs (25 mg). To guarantee complete absorption of the substances by the membranes and to establish functionalization between the Ag and the BC hydroxyls, the membranes were manually pressed with absorbent paper to remove excess water to improve the absorption of the AgNPs after being immersed in the corresponding solutions for 24 h, with rotation every 2 h, and a hot plate magnetic stirrer at 50 °C (Fischer et al. 2017).

### Characterization

An alteration in color was visually detected in the filtrate devoid of cells after incubation with AgNO₃ for a specific duration. The conversion of Ag^+^ to Ag^0^ occurred through the utilization of a 2-mL aliquot of the aqueous solution at various time points. The UV‒visible spectra were examined at wavelengths ranging from 300 to 600 nm via ultraviolet‒visible (UV‒Vis) spectrophotometry (JENWAY 7315, UK). Three technical replicates were employed for data collection, which were then averaged with the optical density values. TEM images of the AgNPs were captured at the Electron Microscopy Unit, University of Assiut, utilizing a JEM100CX11 instrument operating at 100 kV with a resolution of 0.23 nm (Japan, Tokyo). Carbon-coated 0.3 mm grids were loaded with 7 μL of the formed AgNPs, which were subsequently kept in a Petri dish and allowed to dry at room temperature for 24 h. The NP size distribution was determined by measuring 60 particles and statistically analyzed via the *Gaussian* model for linear fit. Thus, a histogram depicting the NP distribution was produced via the Origin software (OriginLab Corporation 2025, Northampton, MA, USA). The structural properties of the AgNPs, BC, and BC-AgNPs were assessed via X-ray diffraction (XRD) via a D8 Advanced Bruker. XRD patterns were obtained at 2*θ* angles ranging from 10 to 80° with a step size of 0.2. FTIR analysis was conducted on fungal extra-enzymes, AgNPs, KC, and AgNPs@KC to identify organic functional groups. FTIR spectra were recorded via ALPHA II in attenuated total reflection mode from 4000 to 500 cm^−1^ with a resolution of 4 cm^−1^. To evaluate the microstructural characteristics of membrane surfaces, micrographs were taken via a JEOL scanning electron microscope (model JSM-6390LV). Samples were sputter-coated with a 10-nm gold layer using a JEOL fine coater (Model JFC-1600) to ensure optimal conductivity while preventing charge accumulation, following manufacturer protocols for nanomaterial imaging (JEOL Ltd., Tokyo, Japan).

### Antimicrobial properties of the AgNPs@KC nanocomposite materials

Gram-positive bacteria (*Bacillus cereus* ATCC 14579 and *Bacillus subtilis* ATCC 6633) and Gram-negative bacteria (*Escherichia coli* ATCC 25922, *Klebsiella pneumoniae* ATCC 13883, *Salmonella enterica* ATCC 14028, and *Pseudomonas aeruginosa* ATCC 27853) were among the twelve harmful pathogens included in the antimicrobial experiments. In addition to opportunistic fungi (*A. flavus* KY609551, *A. fumigatus* MT994683, *A. niger* KY609552, and *A. terreus* MF582635), pathogenic fungi such as *Candida albicans* (AUMC 13507) and *Candida glabrata* (AUMC 13502) were used for antifungal assessment. The bacterial strains were cultivated on nutrient agar (NA) media, whereas the fungi were propagated on Czapek’s (CzA) and Sabouraud media (SDA). Sample pellicles of the AgNPs@KC nanocomposites were subsequently excised into discs. Following Pfaller et al. (2008), a disc diffusion assay assessed AgNPs solution (10, 20, 40, 60, 80, and 100 µg L^−1^) were applied against tested microbial cultures, demonstrating dose-dependent activity via inhibition zone diameters. To assess the AgNPs@KC nanocomposite’s effect, AgNPs@KC nanocomposite pellicles were cut into 8-mm discs. These discs aseptically transferred onto inoculated agar surfaces to ensure uniform contact for antimicrobial testing. The NA, CzA, and SDA plates were incubated at 37 °C to facilitate optimal diffusion of the AgNPs@KC nanocomposites to the surfaces of the bacterial and fungal cells. Following an incubation period of 24 h for bacteria and 72 h for fungi, inhibition zones surrounding the discs were observed, and their diameters were quantified. Cefotaxime (20 μg), fluconazole, and kombucha cellulose membranes were used as positive and negative controls, respectively.

### Statistical analysis

SigmaXL ver. 10 was used for the design of experiment (DOE) (SigmaXL Inc., Ontario, Canada). The Origin software ver. 2025 (OriginLab Corporation, Northampton, MA, USA) was used to analyze the acquired duplicate datasets via two-way ANOVA (Tukey design), with a *p*-value of ≤ 0.05 considered to indicate statistical significance.

## Results

### Synthesis and properties of the mycogenic AgNPs

**Fig. 1:**
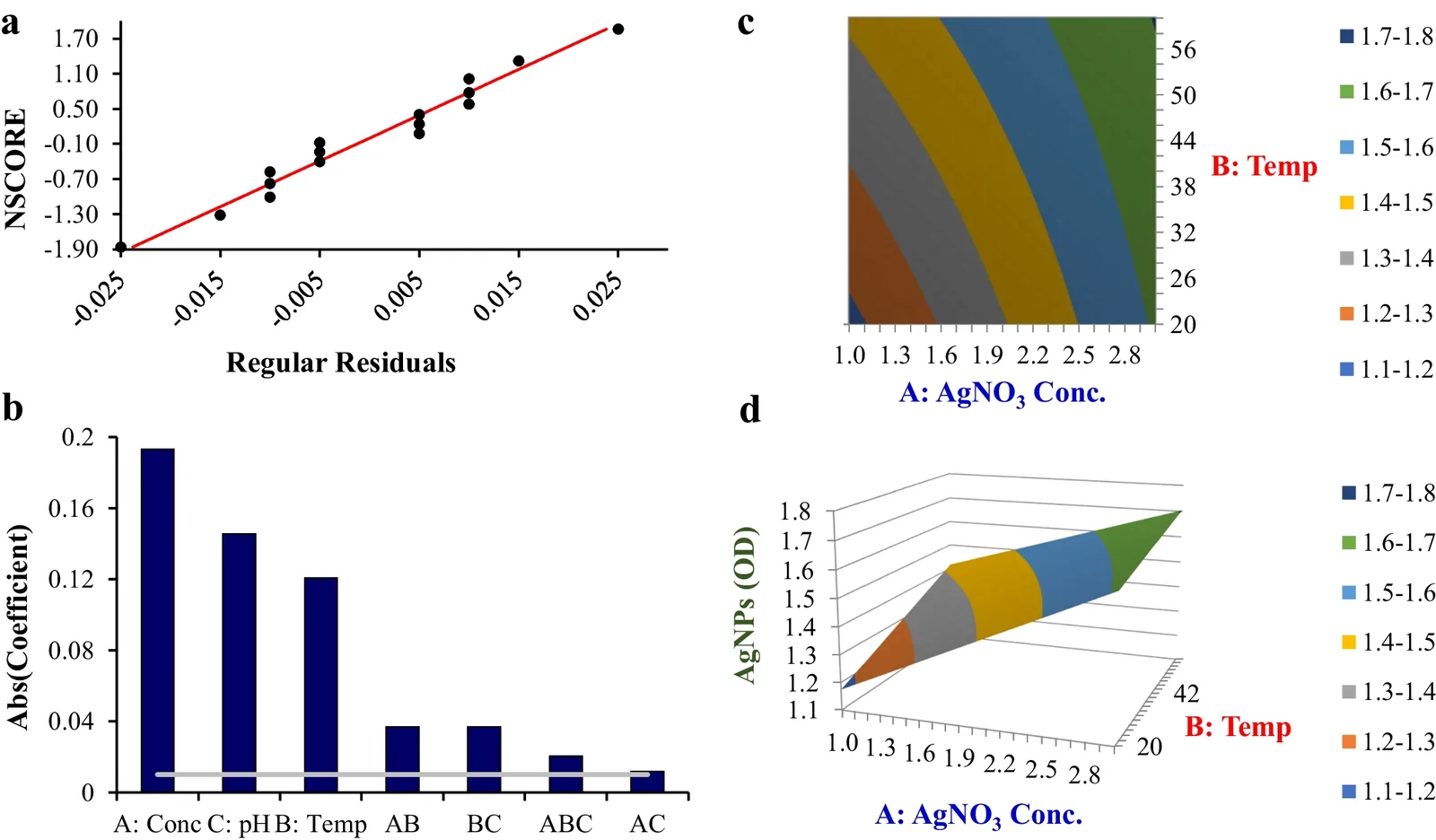
DOE statistical analysis charts for the generation of AgNPs. **a** Chart of the normal probability of regular residuals of AgNPs production, **b** Pareto chart of coefficients, **c** DOE contour chart, and **d** DOE 3-dimensional surface chart

The fungal isolate, *Aspergillus fumigatiaffinis* PP235788.1 (Obiedallah et al. 2024), demonstrated high efficiency in AgNP synthesis, exhibiting a yellowish-brown hue at 6 h, which intensified until 48 h, indicating AgNP formation. After that, the colloidal AgNPs were confirmed via UV‒visible spectroscopy (Online Resource 1, Supplementary Fig. 1; Fig. 1, inset).

An absorbance peak at approximately 425 nm corresponds to the collective oscillations of AgNP conduction electrons, known as localized surface plasmon resonance (LSPR). The UV–visible spectrum remained stable for 3 weeks without peak shifts, indicating the specificity of the AgNPs, with the peak intensity correlating to the quantity of AgNPs generated.

### Optimized parameters for improving AgNP synthesis

The DOE multiple regression model was defined by the following equation:

$$OD\:=\:1.330625\:+\:0.193125\ast A(Conc)\:+\:0.120625\ast B(Temp)\:+\:0.145625\ast C(pH)\:-\:0.036875\ast AB\:-\:0.011875\ast AC-\:0.036875\ast BC\:+\:0.020625\ast ABC$$

The model demonstrated an excellent fit (*R*^2^ = 99.80%, *p* < 0.05) with a standard deviation of 0.0175 (Supplementary Table 1 (Online Resource 1)). The DOE analysis identified pH and AgNO₃ concentration as critical factors. Figure 1a (normal probability plot) confirms residual normality, while Fig. 1b (Pareto chart) ranks parameter significance. Figures 1** c** (contour plot) and **d** (3D surface plot) visualize the optimal AgNP synthesis region at pH 8 and 2 mmol L^−1^ AgNO₃. The contour and 3D surface plots revealed the optimal conditions of a 2-mmol L^−1^ AgNO₃ concentration, pH 8, and 60 °C, resulting in a response of 99.80.

**Fig. 2:**
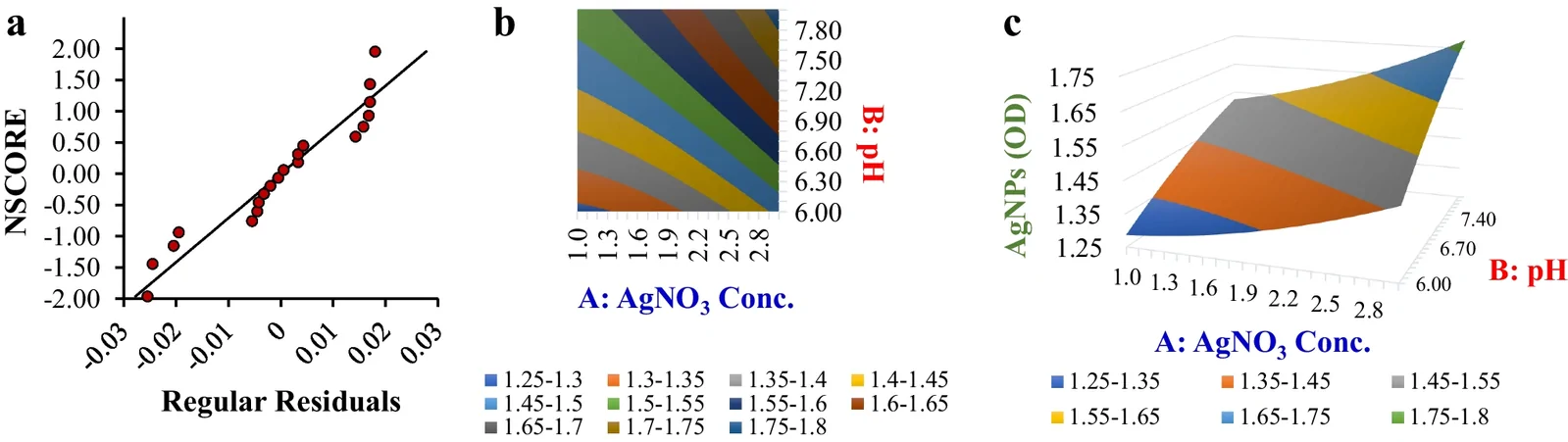
Response surface methodology (RSM) for optimizing AgNPs production. **a** Probability plot for residual normality; **b** two-dimensional contour map of response variables showing the interaction between the AgNO₃ concentration (A) and pH (B); **c** 3D surface plot illustrating the response surface for AgNPs production (OD) as a function of the AgNO₃ concentration (A) and pH (B)

Response surface methodology (RSM) was used for optimization when the pH and AgNO₃ concentration were determined to be key parameters. The ideal center point was determined by a central composite design (CCD) at pH 8.0 and an AgNO₃ concentration of 2 mmol L^−1^ (Fig. 2). The RSM regression model was defined as follows: AgNPs (OD) = 1.502713043 + 0.1075 * A (Conc.) + 0.13625942 * B (pH) + 0.01875 * AB + 0.0321 * AA − 0.021621739 * BB.

The contour and 3D surface plots (Fig. 2) illustrate the relationships between the factors and response, whereas the normal probability plot confirms the model’s predictability. The ANOVA results (Supplementary Table 2 (Online Resource 1)) demonstrate the model’s statistical significance, with a high *R*^2^ value and a *p*-value that is significant (< 0.05).

**Fig. 3:**
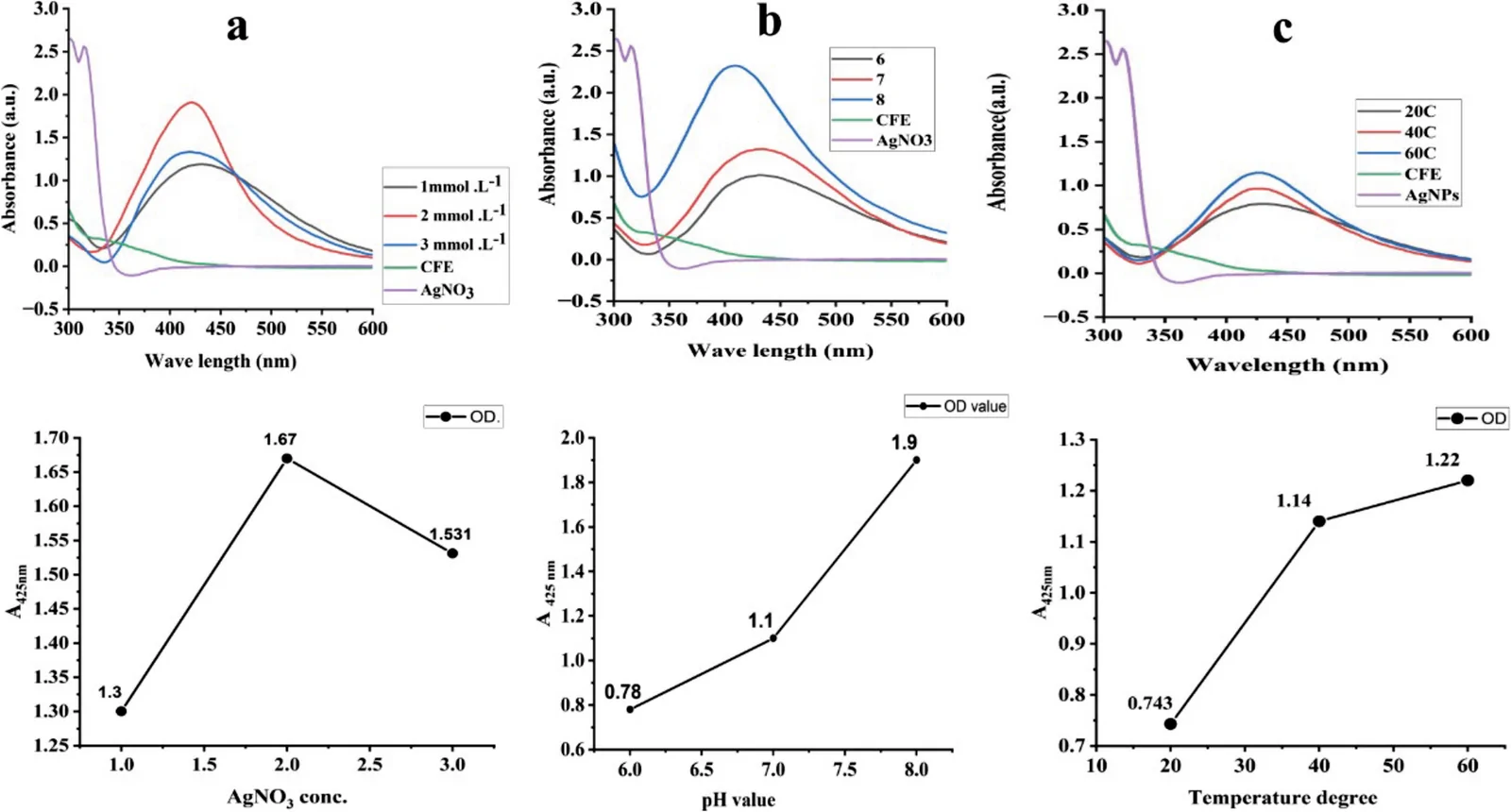
UV‒visible spectra of AgNPs at various AgNO₃ concentrations (**a**), pH values (**b**), and temperatures (**c**) after 24 h. The insets show Linear curves of the OD at 425 nm

The optimized conditions for AgNPs biosynthesis—AgNO₃ (2 mmol L^−1^), 60 °C, and pH 8—were further validated through UV‒visible spectra and linear charts. The UV–visible spectra (Fig. 3a) exhibited a distinct peak at 425 nm, characteristic of AgNPs, confirming successful synthesis under the optimized conditions. The linear chart (Fig. 3b) revealed a strong correlation between AgNP production (OD) and reaction time, with a rapid increase in optical density within the first 2 h at 60 C, followed by stabilization. These results underscore the efficiency of the optimized conditions in enhancing AgNP production and reducing the synthesis time.

**Fig. 4:**
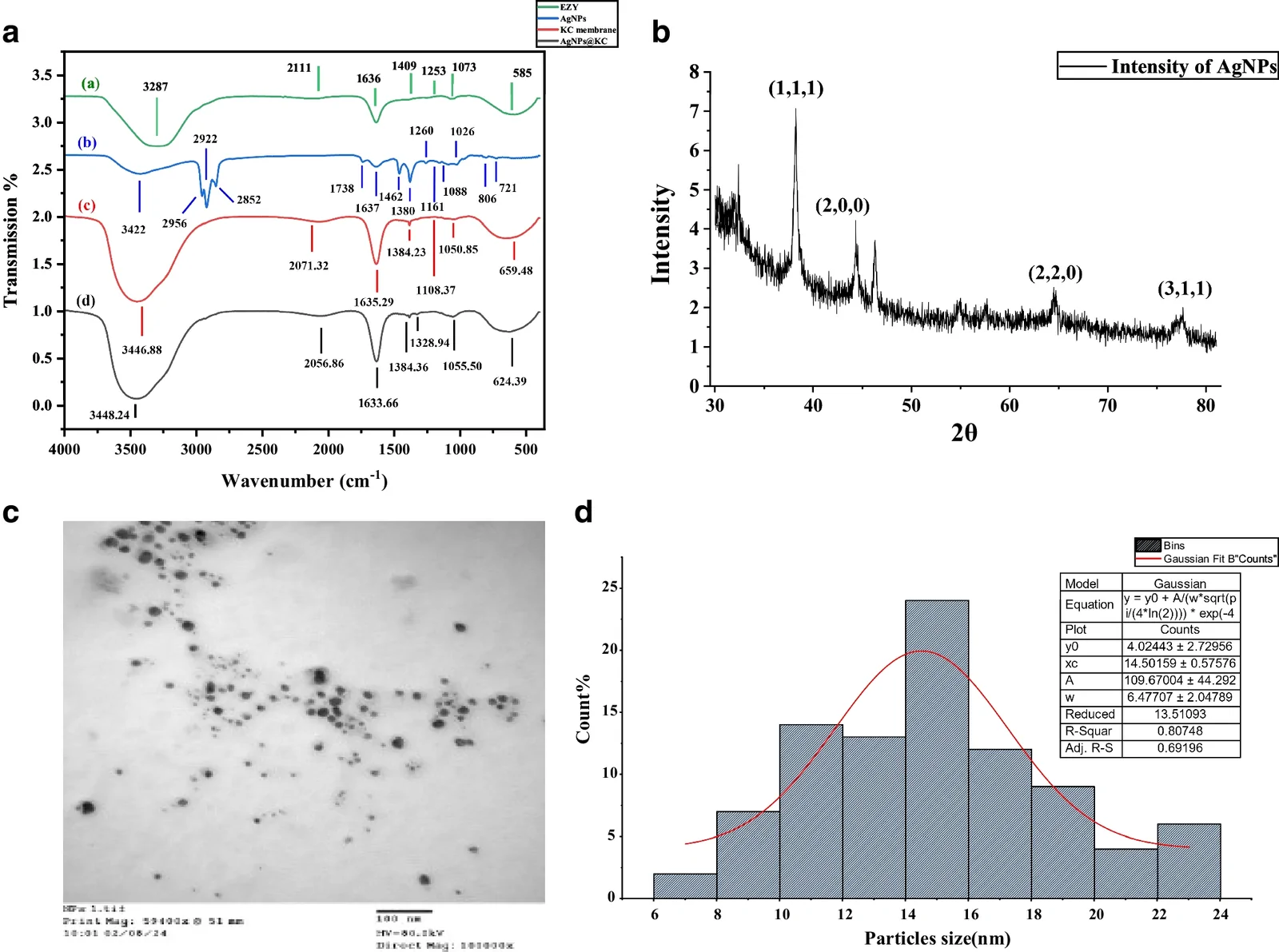
Characterization of AgNPs synthesized using extracellular fungal filtrate of *A*. *fumigatiaffinis*: **a** FTIR spectra of fungal filtrate, AgNPs, pure KC, and AgNPs@KC nanocomposites; **b** XRD patterns with indexed lattice planes (111), (200), (220), and (311) of face-centered cubic (FCC) Ag; **c** TEM micrograph; **d** size distribution histogram (Gaussian fit; average size: 14.5 ± 0.57 nm)

The impact of fungal filtrate phytochemicals on AgNP synthesis and stability was analyzed via Fourier transform infrared (FTIR) spectroscopy (Fig. 4a). The fungal enzyme spectrum revealed bands at 3287 cm^−1^ (–OH stretching), 2111 cm^−1^ (C–H stretching), 1636 cm^−1^ and 1409 cm^−1^ (C = O stretching), 1235 cm^−1^ (CH₃ bending), and 1073 cm^−1^ (C–O and C–N stretching). After the reaction with AgNO₃ (Fig. 4a), peaks at 3421.88 cm^−1^ (N–H stretching), 2955 cm^−1^ (C–H stretching), 1738.29 cm^−1^ (C = O stretching), and 1637.95 cm^−1^ (N–H stretching) were observed, along with minor peaks at 806.85 cm^−1^ (C = N stretching). The shift from 3287 cm^−1^ (–OH) to 3421.88 cm^−1^ (N–H) and the appearance of 1637.95 cm^−1^ (N–H) confirm protein-mediated Ag⁺ reduction, as NH groups in fungal enzymes bind and stabilize nanoparticles (Irfan et al. 2025). The retained C = O peaks (1738.29 cm^−1^, 1636 cm^−1^) suggest polysaccharides also contribute to capping, while the new C = N peak (806.85 cm^−1^) indicates imine formation during synthesis, a hallmark of fungal-derived AgNPs (Irfan et al. 2025).

The FTIR spectrum of dried KC revealed characteristic peaks of bacterial cellulose (BC) (Fig. 4a), including a prominent band at 3446.88 cm^−1^ for O–H stretching, 1635.29 cm^−1^ and 1384.23 cm^−1^ for C–H bond deformation and 1161 cm^−1^ for C–O–C glycosides. The functional groups in the AgNPs@KC nanocomposite correspond to both the crystalline KC and the AgNPs (Fig. 4a). Notable peaks at 1633.66 cm^−1^, 1055.50 cm^−1^, and 624.39 cm^−1^ are indicative of the KC composition. Additionally, the peaks at 3448.24 cm^−1^, 2056.86 cm^−1^, and 1633.66 cm^−1^ shifted because of the reduction process involving AgNO₃ and *A. fumigatiaffinis* cell filtrate. The FTIR spectra of pristine KC and AgNPs@KC nanocomposite were similar, indicating that the distribution of Ag through physical bonding to KC nanofibers did not significantly disrupt the KC network.

XRD analysis of the synthesized AgNPs (Fig. 4b) revealed four prominent diffraction peaks at 2*θ* = 38.00°, 44.28°, 64.85°, and 77.44°, corresponding to the (111), (200), (220), and (311) planes of face-centered cubic (FCC) metallic silver (JCPDS 04–0783). The average crystallite size, calculated using the Scherrer equation (*D* = *Kλ*/*β*cos*θ*) for the (111) peak (*β* = 0.45°), was 15.2 ± 0.8 nm, consistent with TEM measurements (14.50 ± 0.58 nm). The peak sharpness and absence of broadening confirm high crystallinity.

TEM images revealed that the synthesized AgNPs exhibited a nearly spherical morphology (Fig. 4c), which was consistent with the UV‒visible spectral data. The diameters of the AgNPs ranged from 6 to 23 nm, with an average size of 14.50 ± 0.58 nm, as illustrated in the particle size distribution histogram (Fig. 4d). The Gaussian function used is a standard form commonly applied in statistical modeling (Rice 2007).

### Production and properties of AgNPs@KC nanocomposites

**Fig. 5:**
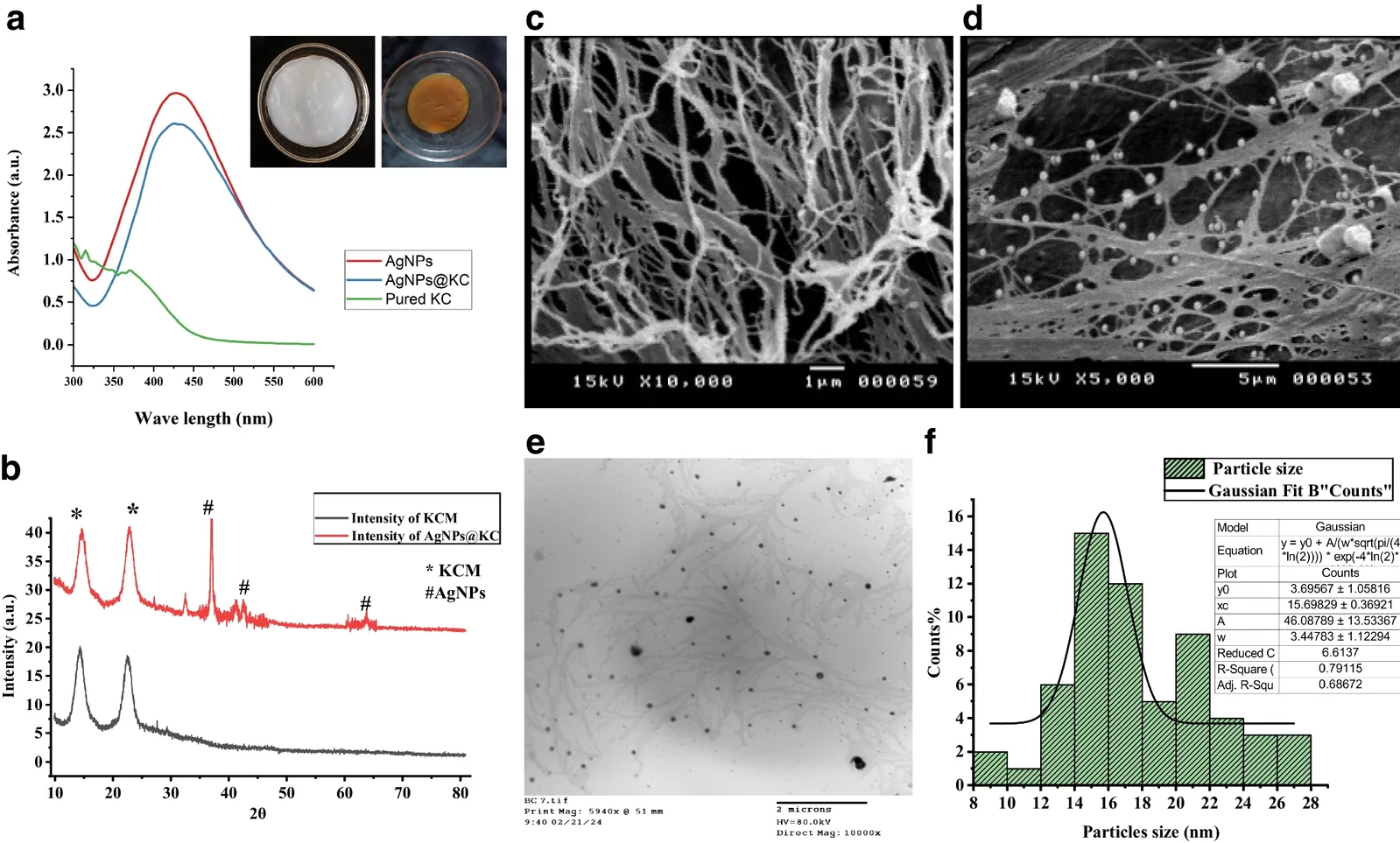
Characterization of formed AgNPs@KC. **a** UV–visible spectra of KC (control) and AgNPs@KC. Insets: color changes due to AgNPs absorption. **b** XRD patterns of pure KC and AgNPs@KC nanocomposites; the Bragg reflection planes of the respective crystallites are labelled. **c** SEM micrograph of the pure KC membrane. **d** SEM micrograph of AgNPs@KC nanocomposites. **e** TEM micrograph showing the spherical morphology. **f** Gaussian-fit size distribution profile of the AgNPs; the average size distribution is 17.88 ± 0.36 nm

The pellicles of KC samples were treated with H₂O₂ to eliminate symbiotic bacteria and yeast, yielding purified white pellicles (Fig. 5a**, **inset). The AgNPs@KC nanocomposite was synthesized through a wet process involving the impregnation of AgNPs into purified KC. The incorporation of AgNPs darkened the color of the KC, resulting in a yellowish-brown hue due to the integration of AgNPs into the KC matrix (Fig. 5a, inset).

Unlike the transparent KC without AgNPs, the AgNPs@KC samples presented a distinct absorption peak at approximately 440 nm and ~ 3 O.D., confirming the successful incorporation of AgNPs (Fig. 5a).

Scanning electron microscopy (SEM) images of the KC and AgNPs@KC nanocomposite sheets showed that the pure KC nanofibers formed interconnected and dense films with irregular pores (Fig. 5c). In contrast, the AgNPs@KC nanocomposite sheets (Fig. 5d) revealed the distribution of AgNPs on the KC fibrils and their particle size. As the AgNP content increased, the number of AgNPs on the composite surfaces also increased, with the voids between KC nanofibrils being occupied by AgNPs. Individual spherical AgNPs trapped among the KC nanofibrils were observed.

Transmission electron microscopy (TEM) images of the AgNPs integrated with kombucha cellulose (KC) with histograms of the particle size distribution analyzed via a Gaussian fitting (Fig. 5 e and f) showed that the size of the spherical AgNPs formed via hydrothermal reduction ranged from 11 to 40 nm, with an average size of 17.88 ± 0.36 nm.

The XRD pattern of the AgNPs@KC nanocomposite (Fig. 5b) shows peaks at 14.58° and 22.7°, confirming the presence of crystalline cellulose I in the KC membrane, which is associated with the (100) and (110) planes of cellulose Iα. The diffraction peaks at 38.2° (111), 46.3° (200), and 64.5° (202) indicate reflections from the crystal planes of the AgNPs.

### Antimicrobial activity

AgNPs demonstrate superior effectiveness against Gram-negative bacteria relative to Gram-positive strains. Antimicrobial assays indicated concentration-dependent inhibition, with Gram-positive and Gram-negative bacteria (*B. subtilis* ATCC 6633 and *E. coli* ATCC 25922 showing 34.33 ± 1.2 and 36.1 ± 0.4 mm, respectively, at 100 µg/mL) exhibiting heightened sensitivity compared to other bacterial strains. *Klebsiella pneumoniae* ATCC 13883 (23 ± 1.1 mm) and *Salmonella enterica* ATCC 14028 (27.3 ± 0.6mm) displayed moderate sensitivity at the maximum concentration tested. Fungal responses were notably diverse, with *Aspergillus* spp. (*A. niger* KY609552: 25.7 ± 0.3 mm at 100 µg/mL) demonstrating higher susceptibility, whereas *Candida* spp. exhibited resistance at concentrations below 60 µg/mL (*C. glabrata* AUMC 13502: 20.6 ± 0.4 mm at 100 µg/mL) **(**Online Resource 1, Supplementary Fig. 2 & 3; Supplementary Table 3**)**.

**Fig. 6:**
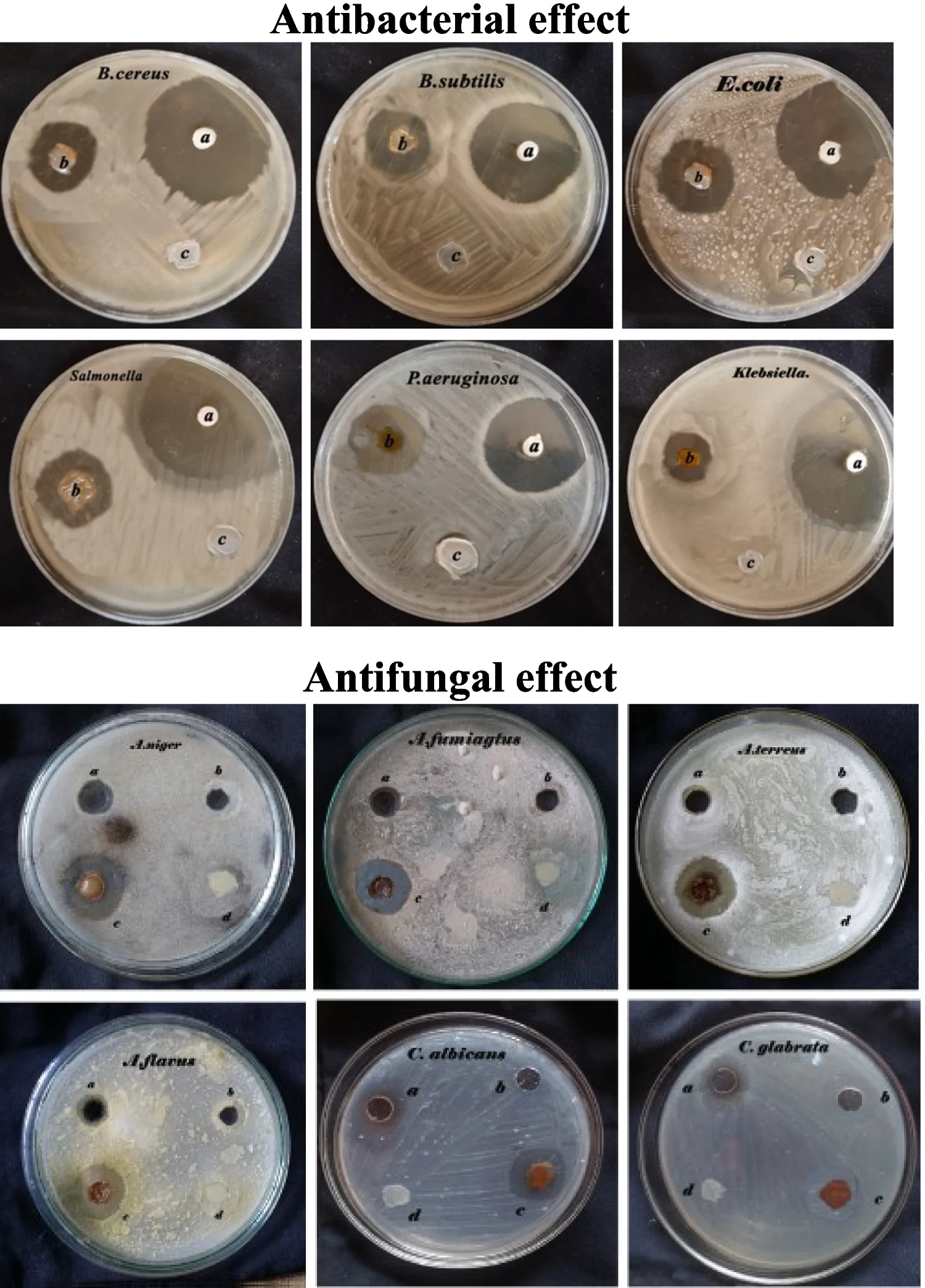
Antimicrobial activity of the AgNPs@KC nanocomposites. Antibacterial effect: **a** positive control (cefotaxime), **b** AgNPs@KC nanocomposites, and **c** pure KC membrane. Antifungal effect: **a** MIC of AgNPs (10 μg L^−1^ in case of *Aspergillus* isolates and 60 μg L^−1^ in *Candida* isolates), **b** negative control (AgNO₃), **c** AgNPs@KC nanocomposites, and **d** pure KC membrane

This study also focused on evaluating the antimicrobial properties of AgNPs@KC nanocomposites against various pathogenic Gram-negative and Gram-positive bacteria via the disc diffusion method **(**Online Resource 1**, **Supplementary Table 4) (Fig. 6**)**. The control group (pure KC) presented no inhibition zone at the center of the plate, whereas the AgNPs@KC nanocomposites presented clear inhibition zones, confirming their antimicrobial activity.

**Fig. 7:**
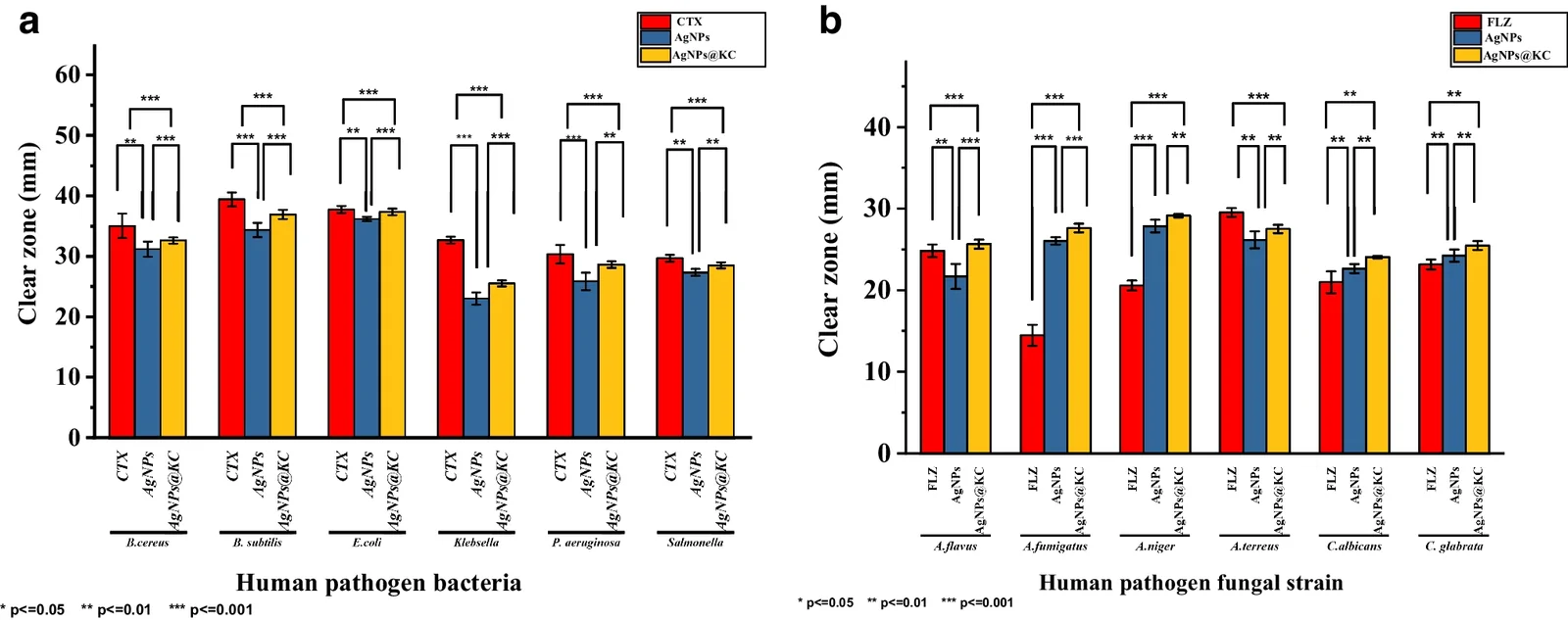
Tukey post hoc analysis of the AgNPs@KC nanocomposites against human pathogenic microorganisms. **a** Antibacterial activity and **b** antifungal activity

The greatest inhibition zones were recorded for *B*. *subtilis* ATCC 6633 (34.55 ± 0.76 mm) and *E*. *coli* ATCC 25922 (37.26 ± 0.55 mm). Tukey post hoc analysis revealed that *E*. *coli* had the most significant mean clear zone (37.26 mm), which was markedly different from those of the other tested bacterial groups (*p* < 0.001) (Fig. 7a). In contrast, *Klebsiella* pneumoniae ATCC 13883 exhibited the smallest mean clear zone (25.5 mm) and showed significant differences in zone size compared with the other bacterial species (Fig. 7a).

The AgNPs@KC nanocomposites were evaluated as antifungal agents against aspergillosis-causing fungi (Online Resoource1**, **Supplementary Table 4) (Fig. 7b). The largest inhibition zone was observed for *A*. *niger* KY609552 (29 ± 0.2 mm), followed by *A*. *fumigatus* MT994683 (27.6 ± 0.52 mm). Tukey post hoc analysis revealed that *A*. *niger* had the most significant mean clear zone (29 mm), differing markedly from the other fungal groups (*p* < 0.001) (Fig. 7b). In contrast, *C*. *albicans* presented the smallest mean clear zone (24 mm), indicating moderate significance compared with the positive control (*p* < 0.01).

Notably, AgNPs@KC composites outperformed free AgNPs in all assays, highlighting the synergistic role of the kombucha cellulose matrix.

## Discussion

This study introduces a novel approach involving the combination of myco-synthesized AgNPs with the kombucha SCOBY cellulose membrane, a combination that has not been previously reported. Unlike previous studies that focused on chemically synthesized AgNPs or other bacterial cellulose sources. This unique combination not only enhances the stability of AgNPs but also provides a sustainable and biocompatible platform for antimicrobial applications. Thus, we used *Aspergillus fumigatiaffinis* PP235788.1, previously isolated from *Artemisia judaica* (Obiedallah et al. 2024), to highlight the potential of endophytic fungi for the green synthesis of nanomaterials, where the formation of AgNPs was evidenced by a brown hue in the reaction mixture. Kowshik et al. (2002) noted that the reduction of Ag^+^ to Ag^0^ is indicated by the color change of the solution changing to brown, which is attributed to SPR excitation of the AgNPs. Vahabi et al. (2011) reported that a reddish tint in biomass solutions signifies AgNPs formation via *Trichoderma reesei*. The coloration of AgNPs can be detected through SPR absorbance, which involves the oscillation of conductive electrons upon light exposure (Slistan-Grijalva et al. 2005). The green synthesis method is preferred over traditional techniques because of its environmental benefits, cost-effectiveness, scalability, and ability to avoid harsh conditions (Bhambure et al. 2009). Subsequent studies have confirmed that color change is the primary visual cue for NP synthesis (Bhambure et al. 2009; Kalimuthu et al. 2009; Du et al. 2011; Verma et al. 2011; Prusinkiewicz et al. 2012; Soni & Prakash 2012; Gupta & Bector 2013; Priyadarshini et al. 2014; Vala 2015). The UV–visible spectrum exhibited a distinct peak at 425 nm, characteristic of spherical AgNPs due to dipole plasmon resonance, where narrower peaks (< 50 nm FWHM) indicate monodispersity (Kelly et al. 2003; Yang et al. 2012). This aligns with TEM observations of spherical morphology. The fungal filtrate exhibited a yellowish-brown hue at 6 h, intensifying until 48 h (Supplementary Fig. 1), indicating progressive AgNPs formation. This timeframe (6–48 h) is notably faster than reports for other *Aspergillus* spp. (e.g., 72–120 h for *A*. *niger*, Ahmad et al. 2003) and comparable to efficient reducers like *Fusarium oxysporum* (24–48 h, Birla et al. 2013), highlighting *A*. *fumigatiaffinis'* rapid metal reduction capability.

Temperature, pH, and synthesis duration are species-specific factors that can be adjusted to achieve desired nanoparticle characteristics. Modifying parameters for fungal cultivation and synthesis is essential for controlling nanoparticle size and morphology (Birla et al. 2013). According to previous studies, nanoparticles with a variety of morphological and physicochemical characteristics can be produced by manipulating variables such as temperature, pH, culture medium, biomass quantity, and metal precursor concentration (Birla et al. 2013; Rajput et al. 2016; Saxena et al. 2016; Liang et al. 2017; Aboul-Nasr et al. 2021). The reaction velocity reported in our study is directly proportional to the silver ion concentration, with higher levels enhancing fungal nanoparticle production, where the synthesis of nanoparticles was faster and more stable at a concentration of 2 mmol^−1^. Spectrophotometric analysis revealed that lower concentrations yielded broader peaks at 435 nm, indicating larger AgNPs sizes. Stability assessments revealed that a concentration of 2 mmol L^−1^ maintained optimal stability for more than 3 months. This is Linked to supersaturation During nucleation, which increases the nucleation rate by approximately 10–70% (Thanh et al. 2014). These results correspond with findings of Ahmad et al. (2003) and Birla et al. (2013), who reported peak AgNPs production at 1.5 mmol^−1^.

The reduction of metallic ions is notably affected by the hydrogen ion concentration, which influences the nanoparticle size and shape during synthesis, as demonstrated by Drexler et al. (1991). Our findings indicate that the introduction of silver salt reduced the pH to 6.5, indicating the onset of silver nanoparticle synthesis. A pH range of 6.5–8.5 significantly reduced the synthesis duration and enhanced the AgNPs yield, supporting prior research on alkaline ions necessary for metal ion reduction (Mohanpuria et al. 2008). Nayak et al. (2013) noted that nitrate reductase enzyme conformation could be adjusted according to proton concentration, thereby impacting nanoparticle morphology and size. Higher pH leads to increased competition between protons and metallic ions for bonding, enhancing nanoparticle synthesis efficiency under alkaline conditions, as reported by Sintubin et al. (2009). A relatively high pH shortened the synthesis time and increased the size distribution and polydispersity index of the nanoparticles, according to Aboul Nasr et al. (2021) and Du et al. (2015). According to Gurunathan et al. (2009), these characteristics improve stability via electrostatic repulsion among dispersing anions. The results of this study revealed that the faster reactions were induced with higher pH values. Azmath et al. (2016) reported that *Colleotrichum* sp. synthesized AgNPs more rapidly at alkaline pH, completing reactions in 20 min. Conversely, acidic conditions resulted in no Ag^+^ reduction, which aligns with the findings of Balakumaran et al. (2015), who reported no color change at pH levels 1 and 4, with color change beginning at pH 6. The current study demonstrated that elevated temperatures led to reduced variability in silver nanoparticle formation. This finding is consistent with that of Birla et al. (2013), who reported increased protein secretion from *Fusarium oxysporum* filtrate at 60 to 80 °C, which was correlated with increased synthesis rates and surface plasmon absorbance. Despite the rapid synthesis of AgNPs at high temperatures, their stability was compromised, with lasting stability noted only at 60 °C for 1 week; this instability was linked to protein denaturation affecting nanoparticle capping. Denaturation hindered Ag^+^ nucleation, causing nanoparticle aggregation and an increase in size, as indicated by Birla et al. (2013). Furthermore, because of decreased enzyme activity throughout the synthesis process, suboptimal temperatures result in larger nanoparticle sizes and poorer stability (Husseiny et al. 2015).

In our investigation, TEM micrographs revealed that the AgNPs synthesized under the optimized conditions displayed a well-dispersed distribution with consistent spherical morphology and varied sizes. The mean diameter of the synthesized AgNPs ranged from 6 to 23 nm, as determined by size distribution. Numerous studies have confirmed that biosynthesized AgNPs exhibit varying dimensions; for example, Jain et al. (2011) reported that *A. flavus* NJP08 produced spherical, monodisperse AgNPs with sizes between 10 and 35 nm. Ammar and El-Desouky (2016) noted that AgNPs from *Penicillium expansum* varied from 14 to 25 nm in size. The FTIR results indicated the presence of –CH and –NH functional groups involved in the reduction process, suggesting that amino acids such as cysteine and methionine are crucial in AgNPs biosynthesis via AgNO_3_ reduction by fungal biomolecules. Our FTIR peaks (1637.95 cm^−1^, 806.85 cm^−1^) closely match fungal AgNPs in Lee et al. (2021), where N–H and C = N groups were critical for stability. However, the stronger C = O signal (1738.29 cm^−1^) in our work suggests enhanced polysaccharide capping, likely due to kombucha’s unique microbial consortium.

Alkaline pH conditions promote hydroxide ion (OH-) predominance, enhancing electrostatic repulsion and preventing particle aggregation. Increased colloidal stability at higher pH levels aids in synthesizing nanoparticles with reduced dimensions (Priyadarshini et al. 2014). X-ray diffraction results revealed peaks aligning with the Bragg’s reflections of the AgNPs, supporting the findings of Shankar et al. (2003). Current findings corroborate previous data on AgNPs synthesis by various bacterial species (Southam & Beveridge 1994; Husseiny et al. 2007), fungi (Mukherjee et al. 2001, 2002), and actinomycetes (Ahmad et al. 2003). The results of this investigation are further validated by findings from Prema and Rincy (2009) and Narasimha et al. (2011).

In this study, a cellulose kombucha disc was used as an example of bacterial cellulose that can be used as a silver composite because of its flexibility and high purity. Furthermore, bacterial cellulose production by certain strains, such as *Komagataeibacter*, is significantly lower than that of SCOBY. Research indicates that the mutual growth promotion between bacteria and yeast in SCOBY enhances BC production. Consequently, SCOBY has been identified as the optimal culture for BC production (Chawla et al. 2009).

The color of the cellulose kombucha membrane changed from colorless to brown during the study because nanopores can exist in a three-dimensional BC network. The BC acts as a nanoreactor during hydrothermal reduction, facilitating the nucleation and development of silver particles through in situ synthesis employing BC as a template (Maneerung et al. 2008; Yang et al. 2012). The abundant hydroxyl groups on BC microfibrils act as anchoring sites for silver nanoparticles, whereas the nanosized pores serve as nanoreactors for AgNP growth and nucleation, influencing the particle size and distribution (Yang et al. 2017). Electrostatic forces are crucial for both the formation and stabilization of silver nanoparticles. In this chemical framework, BC acts as a stabilizer, preventing the aggregation of AgNPs and ensuring uniform dispersion and immobilization. The UV–vis spectra of the AgNPs@KC nanocomposites exhibited a peak at 440 nm, indicative of surface plasmon resonance (SPR) of the spherical or quasi-spherical AgNPs; these findings are consistent with those of Kundu et al. 2009. Li et al. (2015) reported an optical spectrum of AgNPs@KC nanocomposites at 440 nm when a high concentration of silver ions was used with a cellulose membrane. The intensity of the SPR peak was positively correlated with the AgNPs concentration, as indicated by the color changes reflecting increased AgNP content. Furthermore, the broadening of the SPR peak with increasing silver ion concentration suggests a diverse size distribution of the AgNPs (He et al. 2003). This study revealed well-dispersed spherical AgNPs on the KC membrane surface, which was corroborated by previous findings (Kucukcobanoglu et al. 2021). Similarly, Barud et al. (2008) identified shiny AgNPs on BC fibrils linked to silver complex decomposition. Neat BC exhibited a dense, randomly arranged nanofibril network, supporting significant liquid retention, which is consistent with Fischer et al. (2017) and Godinho et al. (2016). Yan et al. (2017) successfully generated BC membranes from *Komagataeibacter hansenii* with comparable fibril sizes. Nevertheless, the high aspect ratio of the nanofibers resulted in unique morphological characteristics. The TEM results align with those of previous studies on AgNPs@BC nanocomposite properties (Shaaban et al. 2023; Yang et al. 2013). The FT-IR spectra of pristine KC and the AgNPs@KC nanocomposite indicated minimal disruption to the BC network due to the Ag distribution. These results agreed with those of Godinho et al. (2016) and Sakai et al. (2006). The X-ray diffraction pattern aligned with earlier findings (Ahmed et al. 2019; Emam 2019; Araújo et al. 2018; Li et al. 2015), showing no shift in the diffraction peak positions after AgNP addition. This suggests a lack of chemical interactions between the AgNPs and BC, which was reaffirmed by further confirmation. Consequently, the potential loss of AgNPs during application should be a critical consideration for antimicrobial materials (Yang et al. 2012). The surface-located Ag particles are prone to loss upon rinsing in saline, highlighting the need for AgNPs to penetrate deeper into the BC matrix to sustain antibacterial efficacy. While this study standardized AgNP loading via a fixed protocol (25 mg AgNPs per membrane), future work could employ TGA or acid digestion-ICP-MS to quantify absolute loading efficiency. However, the consistent antimicrobial efficacy confirms reproducible functionalization.

Recent studies highlight the potential of AgNPs@KC composites in food packaging, where their antimicrobial properties extend shelf life while maintaining biocompatibility (Salama et al. 2021). This aligns with our findings of enhanced stability and antimicrobial efficacy (Fig. 7), suggesting promise for biomedical and industrial applications. While MIC/MBC assays are conventional for soluble antimicrobials, disc diffusion remains the best standard for evaluating immobilized nanocomposites, as it directly reflects surface-mediated microbial inhibition (Barud et al. 2008).

While the AgNPs@KC nanocomposites demonstrated potent antimicrobial activity, potential Ag⁺ leaching—a critical factor for biomedical applications—was indirectly assessed through the stability of inhibition zones over h (Fig. 7). No significant expansion of zones was observed, suggesting minimal ionic leaching under test conditions. This aligns with FTIR data (Fig. 4a) showing strong interactions between AgNPs and KC hydroxyl groups, which stabilize nanoparticles and reduce ion release compared to unbound AgNPs (Yang et al. 2012). However, long-term leaching kinetics should be evaluated in future clinical studies.

The differential efficacy against Gram-negative and Gram-positive bacteria reflects distinct nanoparticle–cell interactions. In Gram-negative bacteria, AgNPs disrupt the outer membrane through LPS oxidation and porin protein denaturation, while in Gram-positive strains, they bind teichoic acids and catalyze peptidoglycan crosslink cleavage (Sidhu et al. 2025). Our results showing greater *E*. *coli* susceptibility (Fig. 7a) align with recent findings that Gram-negative membranes are more vulnerable to nanoscale perforation due to thinner peptidoglycan layers (Sudheer et al. 2022). The sustained antimicrobial activity confirms that AgNPs@KC composites maintain these mechanisms while minimizing ionic leaching. Research indicates that the bactericidal efficacy of nanoparticles is not exclusively linked to the bacterial membrane structure. Consequently, it is impractical to generalize the antibacterial effects of AgNPs on the basis of only a single strain, as bacteria demonstrate diverse sensitivities due to varying cell wall architectures. Nevertheless, several proposed toxicity mechanisms include (a) free silver ions disrupting ATP synthesis and DNA replication; (b) reactive oxygen species generated by ions and AgNPs interacting with sulfhydryl groups, impeding respiration; and (c) direct damage to the cellular membrane by AgNPs, affecting membrane permeability (Tomacheski et al. 2016).

## Conclusion

This research presents a novel method for the biosynthesis of AgNPs via the endophytic fungus *A. fumigatiaffinis* and kombucha SCOBY disc cellulose membrane for AgNPs@KC nanocomposite fabrication via hydrothermal methods. Design of experiments (DOEs) and response surface methodology (RSM) were employed to optimize AgNP synthesis, with optimal conditions achieved at 2 mmol L^−1^ AgNO₃, pH 8, and 60 °C. The formation of the AgNPs@KC nanocomposites was characterized through XRD, FTIR, TEM, and SEM techniques. SEM analysis revealed a uniform distribution of AgNPs, both as discrete spherical particles and as aggregates, within the kombucha SCOBY cellulose matrix. XRD analysis confirmed the incorporation of AgNPs within the AgNPs@KC nanocomposites at approximately 17.88 ± 0.36 nm, which was corroborated by the TEM findings. The AgNPs@KC nanocomposites exhibited significant antimicrobial efficacy against both Gram-negative and Gram-positive bacteria, as well as human pathogenic fungi. These results demonstrate the successful combination of mycosynthesized AgNPs with kombucha SCOBY-derived cellulose membranes, establishing an innovative platform for antimicrobial applications. This hybrid system exhibits exceptional purity and unique physiological properties, distinguishing it from conventional approaches.

## Supplementary Information

Below is the link to the electronic supplementary material.

ESM1 (DOCX 1.39 MB)

## Data Availability

All data generated or analyzed during this study are included in this published article
