# Supplementary material for: Optimized biosynthesis of bioactive silver nanoparticle-kombucha cellulose nanocomposites for enhanced antimicrobial applications
Source: Appl Microbiol Biotechnol. 2025 Sep 29;109(1):209. doi: 10.1007/s00253-025-13585-0 (PMC12479647; doi:10.1007/s00253-025-13585-0)
Supplement: Supplementary file 1 — (DOCX 1.39 MB) [file 253_2025_13585_MOESM1_ESM.docx]

**
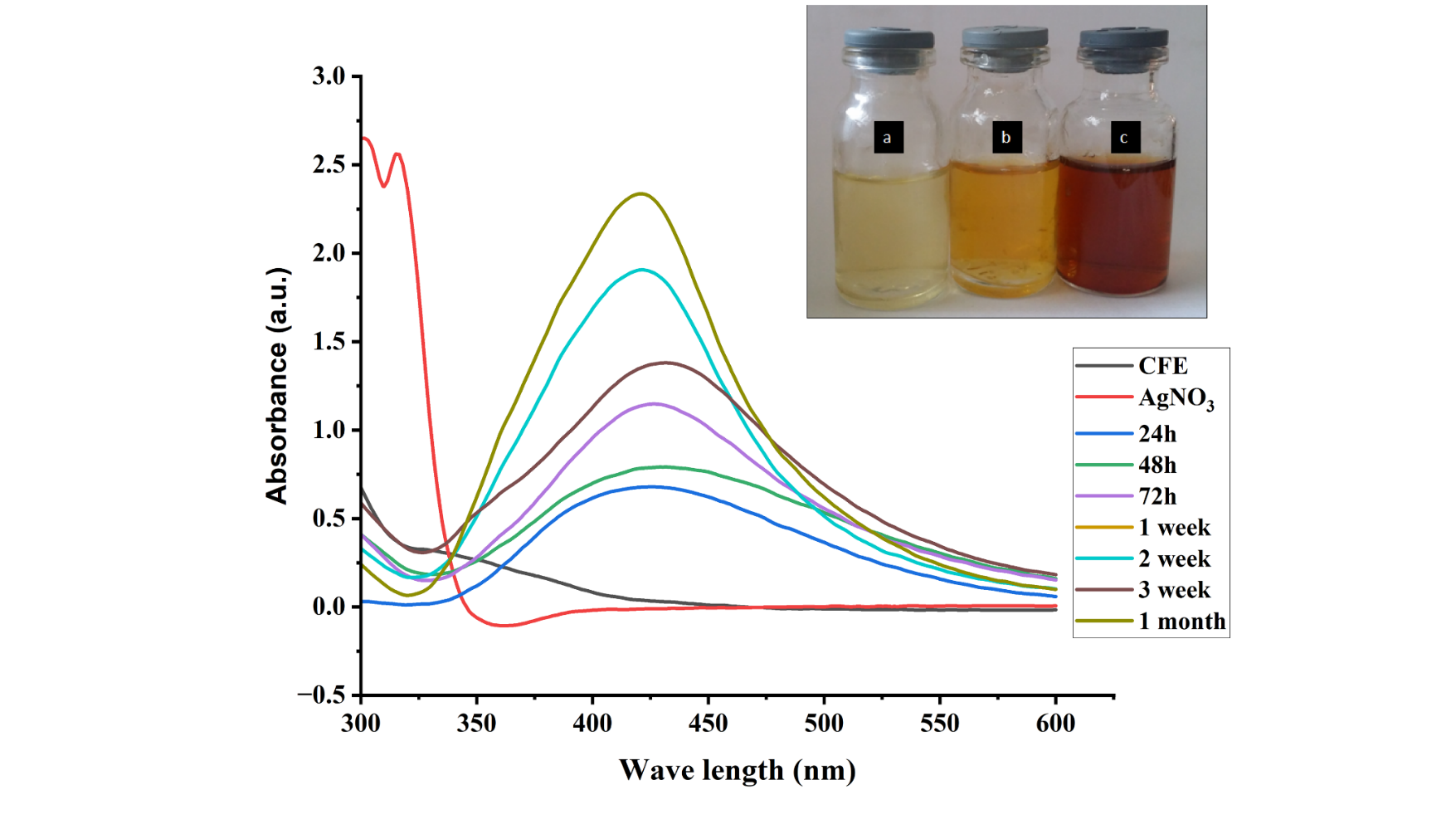
**

**Supplementary Fig. 1** UV–visible spectra of the formed AgNPs after 24 h and up to one month. Inset: Change in color before and after the synthesis of AgNPs. **(a)** Cell-free filtrate of *A. fumigatiaffinis*, **(b)** color of the AgNPs at 6 hr, and **(c)** color intensity increasing to reddish at 24 hr

**
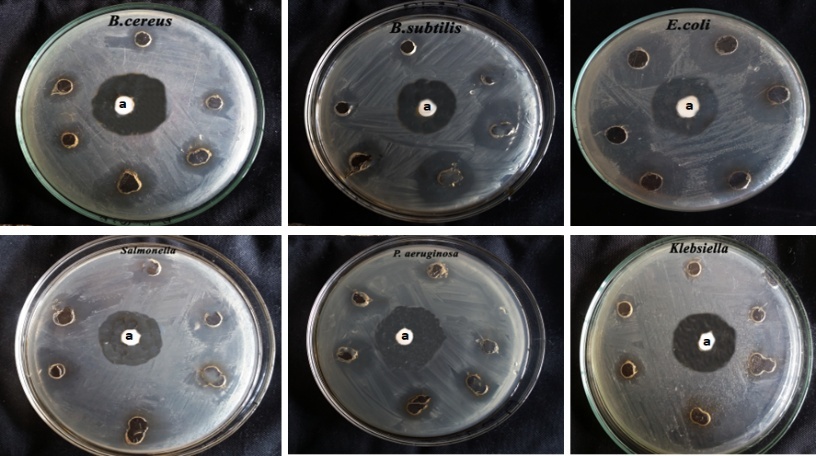
**

**Supplementary Fig. 2** Antibacterial activity of different concentrations of biosynthesized AgNPs produced by extracellular filtrate of *A*. *fumigatiaffinis* against pathogenic bacteria, **(a)** cefotaxime (positive control)


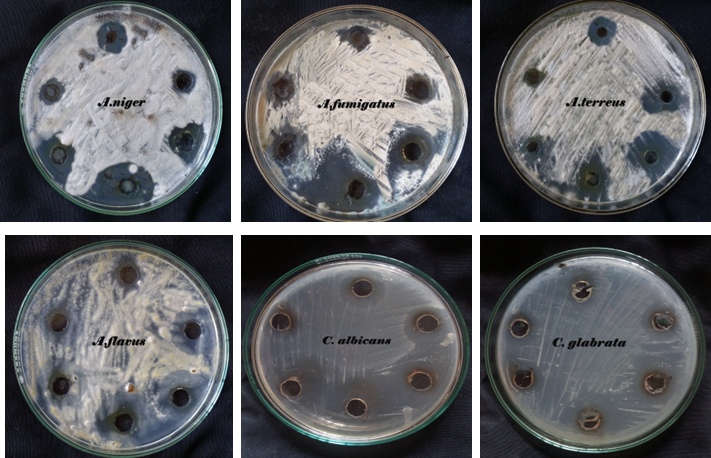


**Supplementary Fig. 3** Antifungal activity of different concentrations of AgNPs synthesized by extracellular filtrate of *A*. *fumigatiaffinis* against pathogenic fungi

**Supplementary Table 1.** Summary of the ANOVA results for the fully factorial design model of AgNP synthesis induced by *A. fumigatiaffinis*.

| **Parameter Estimates (Coded Units):** | | |  | |  | | |  | |  |
| --- | --- | --- | --- | --- | --- | --- | --- | --- | --- | --- |
| **Term** | **Coefficient** | **SE Coefficient** | | **T value** | | **p value** | **VIF** | | **Tolerance** | |
| Constant | 1.33^***^ | 0.004 | | 304.14 | | 0.00 | 1 | | 1 | |
| **A:** AgNO_3_ conc. | 0.19^***^ | 0.004 | | 44.14 | | 0.00 | - | | - | |
| **B:** Temperature | 0.12^***^ | 0.004 | | 27.57 | | 0.00 | - | | - | |
| **C:** pH | 0.15^***^ | 0.004 | | 33.29 | | 0.00 | - | | - | |
| AB Interaction | -0.04^***^ | 0.004 | | -8.43 | | 0.00 | - | | - | |
| AC Interaction | -0.01 | 0.004 | | -2.71 | | 0.03 | - | | - | |
| BC Interaction | -0.04^***^ | 0.004 | | -8.43 | | 0.00 | - | | - | |
| ABC Interaction | 0.02 | 0.004 | | 4.71 | | 0.00 | - | | - | |

**Model Summary:**

| **Metric** | **Value** |
| --- | --- |
| R^2^ | 99.80% |
| Adjusted R^2^ | 99.62% |
| RMSE (Root Mean Square Error) | 0.018 |

**Analysis of Variance for Model:**

| **Source** | **DF** | **SS** | **MS** | **F Value** | **p Value** |
| --- | --- | --- | --- | --- | --- |
| Model | 7 | 1.22 | 0.17 | 569.77 | 0.00 |
| Error | 8 | 0.00 | 0.00 | - | - |
| Pure Error | 8 | 0.00 | 0.00 | - | - |
| Total | 15 | 1.22 | 0.08 | - | - |

*** denotes significance at the 1% level (p < 0.01).
**SE**: standard error, **T**: T value, **VIF**: variance inflation factor.
**DF**: degrees of freedom, **SS**: sum of squares, **MS**: mean square, **F**: F statistic.

**Supplementary Table 2.** ANOVA for the RSM-Based AgNP induction model with *A*. *fumigatiaffinis*.

**Parameter estimates (coded units):**

| **Term** | **Coefficient** | **SE Coefficient** | **T Value** | **p Value** | **VIF** | **Tolerance** |
| --- | --- | --- | --- | --- | --- | --- |
| Constant | 1.50*** | 0.01 | 169.73 | 0.00 | - | - |
| A: AgNO₃ conc. | 0.11*** | 0.01 | 20.98 | 0.00 | 1.00 | 1.00 |
| B: pH | 0.14*** | 0.00 | 27.46 | 0.00 | 1.01 | 0.98 |
| AB Interaction | 0.019* | 0.01 | 2.99 | 0.01 | 1.00 | 1.00 |
| AA Interaction | 0.03** | 0.01 | 3.95 | 0.00 | 1.01 | 0.98 |
| BB Interaction | -0.02* | 0.01 | -2.58 | 0.02 | 1.01 | 0.99 |

**Model Summary:**

| **Metric** | **Value** |
| --- | --- |
| R^2^ | 98.94% |
| Adjusted R^2^ | 98.45% |
| RMSE (Root Mean Square Error) | 0.02 |

**Analysis of Variance for Model:**

| **Source** | **DF** | **SS** | **MS** | **F Value** | **p Value** |
| --- | --- | --- | --- | --- | --- |
| Model | 5 | 0.38 | 0.08 | 241.85 | 0.00 |
| Error | 13 | 0.00 | 0.00 | - | - |
| Lack of Fit | 11 | 0.00 | 0.00 | 3.54 | 0.01 |
| Pure Error | 2 | 0.00 | 0.00 | - | - |

*** denotes significance at the 1% level (p < 0.01), ** at the 5% level (p < 0.05), and * at the 10% level (p < 0.10).

**SE**: standard error, **T**: T value, **VIF**: variance inflation factor.
**DF**: degrees of freedom, **SS**: sum of squares, **MS**: mean square, **F**: F statistic.

**Supplementary Table 3.** Minimum inhibitory concentration (MIC) and zone of inhibition of biosynthesized AgNPs (14 nm) from A. fumigatiaffinis against bacterial and fungal pathogens.

|  |  | | **AgNPs concentration (µg/L^-1^)** | | | | | | |  |
| --- | --- | --- | --- | --- | --- | --- | --- | --- | --- | --- |
|  |  |  | **10** | **20** | | **40** | **60** | **80** | **100** |  |
|  | **Pathogenic bacteria** | | |  | |  |  |  |  |  |
| **1** | *Bacillus cereus* ATCC 14579 | | 9.8±2.9 | 12.9±3.3 | | 16.5±2.2 | 21.2±1.1 | 27.62±4.2 | 31.16± 1.3 | **Diameter of inhibition zones (mm)** |
| **2** | *Bacillus subtilis* ATCC 6633 | 9.8 ± 2.9 | | | 15.4 ±0.9 | 19 ±1.4 | 23 ±2.7 | 30.8 ±1.1 | 34.33± 1.2 |  |
| **3** | *E. coli* ATCC 25922 | 11.8±2.1 | | | 17±2.1 | 22±2.7 | 27.8±2.2 | 33.2±1.6 | 36.1± 0.4 |  |
| **4** | *Klebsiella pneumoniae* ATCC 13883 | 4±1.4 | | | 8.9±1.1 | 11.5±1.4 | 15.8±2.7 | 18.1±2.0 | 23± 1 |  |
| **5** | *Pseudomonas aeruginosa* ATCC 27853 | 3±0.6 | | | 9.4±0.8 | 13.9±1.5 | 18.5±1.4 | 21.4±1.3 | 25.83± 1.4 |  |
| **6** | *Salmonella enterica* ATCC 14028 | 6± 1.4 | | | 11.4±2.1 | 14.4±1.3 | 20.1±1.6 | 23.8±1.6 | 27.3± 0.6 |  |
|  | **Pathogenic fungi** | | | |  |  |  |  |  |  |
| **7** | *A. flavus* KY609551 | 6.7± 0.5 | | | 10.6±0.5 | 15.0±0.1 | 17.6±0.4 | 22.2±0.5 | 23.9±0.5 |  |
| **8** | *A. fumigatus* MT994683 | 8.7±0.6 | | | 11.3±1.2 | 13.0±0.7 | 15.7±0.7 | 21.1±0.5 | 23.3±0.9 |  |
| **9** | *A. niger* KY609552 | 9.8±0.6 | | | 11.8±0.3 | 15.6±0.8 | 20.2±0.6 | 23±0.4 | 25.7±0.3 |  |
| **10** | *A.terreus* MF582635 | 6.6±0.4 | | | 9±0.6 | 11.6±0.4 | 14.1±0.4 | 15.8±0.8 | 18.7±0.6 |  |
| **11** | *Candida albicans* AUMC 13507 | 0 | | | 0 | 0 | 10.3±0.4 | 13±0.8 | 16.3±1.4 |  |
| **12** | *Candida glabrata* AUMC 13502 | 0 | | | 0 | 0 | 12.4±0.2 | 17.3±0.2 | 20.6±0.4 |  |

**Supplementary Table 4.** Diameterof inhibition zones (mm) of AgNPs, AgNPs@KC, and positive controls.

| **No.** | **Pathogenic bacteria** | **AgNPs** | | **AgNPs@KC** | **Positive control** |
| --- | --- | --- | --- | --- | --- |
|  |  |  |  |  | **Cefotaxime** |
| **1** | *Bacillus cereus* ATCC 14579 | | 31.16± 1.3 | 32.6 ± 0.5 | 35 ± 2 |
| **2** | *Bacillus subtilis* ATCC 6633 | 34.33± 1.2 | | 34.55 ± 0.8 | 39.33 ± 1.2 |
| **3** | *E. coli* ATCC 25922 | 36.1± 0.4 | | 37.26 ± 0.6 | 37.8 ± 0.6 |
| **4** | *Klebsiella pneumoniae* ATCC 13883 | 23± 1 | | 25.5± 0.5 | 32.6± 0.6 |
| **5** | *Pseudomonas aeruginosa* ATCC 27853 | 25.83± 1.4 | | 28.63± 0.5 | 30.3± 1.5 |
| **6** | *Salmonella enterica* ATCC 14028 | 27.3± 0.6 | | 28.566± 0.4 | 29.6± 0.6 |
|  | **Pathogenic fungi** |  | |  | **Fluconazole** |
| **7** | *A. flavus* KY609551 | 21.66± 1.5 | | 25.63 ± 0.6 | 24.83± 0.8 |
| **8** | *A. fumigatus* MT994683 | 26.04± 0.5 | | 27.6 ± 0.5 | 14.46 ± 1.3 |
| **9** | *A. niger* KY609552 | 27.83± 0.8 | | 29± 0.2 | 20.56± 0.6 |
| **10** | *A.terreus* MF582635 | 26.16± 1.0 | | 27± 0.5 | 29.46± 0.6 |
| **11** | *Candida albicans* AUMC 13507 | 21.66± 1.6 | | 24± 0.2 | 19.96± 1.3 |
| **12** | *Candida glabrata* AUMC 13502 | 22.6± 0.6 | | 25.46± 0.6 | 23.13± 0.6 |
